# An Unusual Pes Anserinus Variant Encountered During Hamstring Autograft Harvest for Anterior Cruciate Ligament Reconstruction: A Case Report

**DOI:** 10.7759/cureus.115013

**Published:** 2026-08-23

**Authors:** Abdul Khokhar, Mohammed Al-Bajari, Gurudatt Sisodia

**Affiliations:** 1 Trauma and Orthopaedics, Manchester University NHS Foundation Trust, Manchester, GBR; 2 Critical Care Medicine, Manchester University NHS Foundation Trust, Manchester, GBR

**Keywords:** anatomical variation, anterior cruciate ligament reconstruction, gracilis, hamstring autograft, pes anserinus, sartorius, semitendinosus

## Abstract

The pes anserinus (PA) is the common anteromedial tibial insertion of the sartorius, gracilis, and semitendinosus tendons. Variations in this region are well documented and may complicate hamstring autograft harvest during anterior cruciate ligament reconstruction. We present a case of a 29-year-old male who underwent elective right knee arthroscopic anterior cruciate ligament reconstruction with partial medial and lateral meniscectomies. During routine hamstring tendon harvest, the gracilis tendon and semitendinosus tendon were identified and harvested. A third tendon-like structure was also encountered just above the pes anserinus. This structure coursed anteriorly up the thigh on limited proximal inspection, but its precise muscular origin could not be definitively established intraoperatively. It was preserved and remained separate from the harvested tendons. The patient had no immediate neurovascular or functional deficit postoperatively and reported improved knee stability and no pain at six-week follow-up. This case highlights an unusual tendon-like structure encountered during hamstring autograft harvest and emphasises the importance of careful identification of anatomical variants before dividing any unrecognised structure.

## Introduction

The pes anserinus (PA) is the common insertion on the anteromedial tibia of three muscles: sartorius, gracilis, and the semitendinosus [1]; the latter two tendons are commonly harvested as autografts for anterior cruciate ligament (ACL) reconstruction [2]. Variations to the PA anatomy are well documented, with accessory bands most frequently arising from the semitendinosus, and less commonly the gracilis tendon [3,4]. In one anatomical study, accessory bands were identified in 94% of semitendinosus tendons (STTs) and 80% of gracilis tendons (GTs) [5]. Such variations can complicate harvest and lead to premature amputation of the tendons if not recognised and dissected intraoperatively [6,7].

The sartorius forms the most superficial component of the PA and must be identified and separated along with its fascia to allow mobilisation of the gracilis and semitendinosus tendons before harvest [6]. Variations involving discrete accessory tendons of sartorius are not well described in the clinical literature. Such variants appear to be uncommon, and their clinical significance lies in the potential for misidentification during hamstring tendon harvest, with inadvertent injury or unnecessary division of an unrecognised structure. We present a case in which an additional tendon-like structure was encountered intraoperatively following confirmed harvest of the gracilis and semitendinosus tendons, raising the possibility of a rare sartorius tendon variant.

## Case presentation

A 29-year-old male underwent an elective right knee arthroscopic ACL reconstruction with partial medial and lateral meniscectomies; the patient had no other medical conditions. The operation was performed two years after a pivot injury resulting in ACL rupture with associated medial and lateral meniscal tears. The patient initially opted for conservative management and later agreed to proceed with surgery due to ongoing instability.

**Figure 1:**
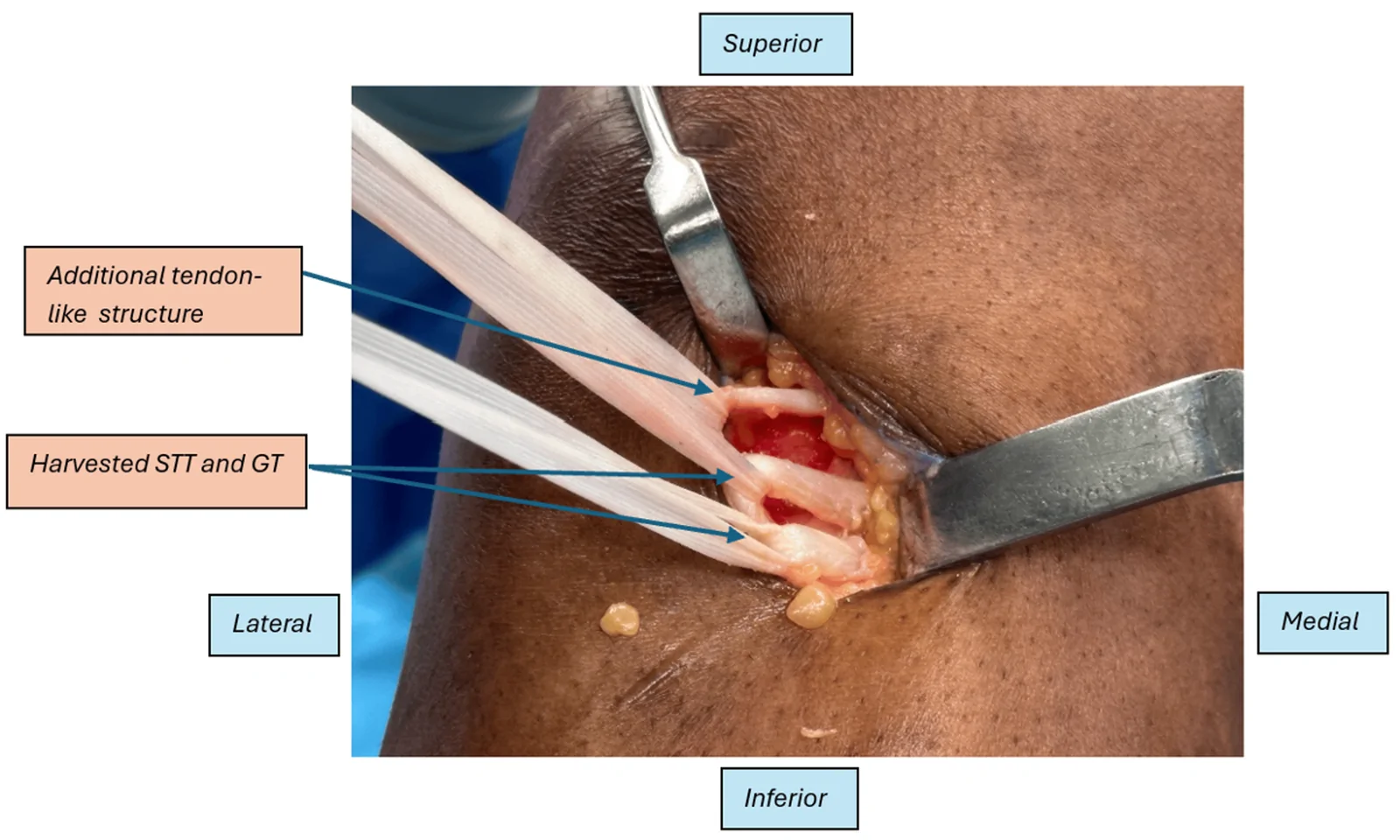
Intraoperative Identification of an Additional Tendon-Like Structure in the Pes Anserinus Region Intraoperative photograph of the right knee pes anserinus (PA) region following harvest of the semitendinosus tendon (STT) and gracilis tendon (GT). The harvested STT and GT are retracted laterally. An additional flat, tendon-like structure is visible within the operative field after dissection through the sartorial fascia. Limited proximal inspection demonstrated that this structure coursed anteriorly up the thigh; however, definitive continuity with a specific muscle belly was not established intraoperatively.

As part of the routine hamstring tendon harvest, the sartorial fascia was incised, and the GT and STT were identified at their anteromedial proximal tibial insertions. A third tendinous structure was also identified superior to the GT and STT, as shown in Figure 1. When followed proximally, this structure coursed anteriorly up the thigh; however, its precise muscular origin could not be definitively established intraoperatively. Further proximal dissection was not undertaken, as this would have required extension of the incision beyond that necessary for the planned procedure and consent for this had not been obtained.

This third structure was preserved. After harvesting the two tendons, the third additional tendon remained in situ and was not connected to the other two tendons that were harvested. The harvested tendons were used as a graft for ACL reconstruction. The procedure was completed without complications.

The patient had no immediate neurovascular or functional deficit postoperatively and reported improved knee stability and no pain at six-week follow-up. At 12-week follow-up, the patient had full active range of motion of the right knee and no pain on movement.

## Discussion

Variations in the anatomy of the pes anserinus (PA) are well documented during hamstring tendon harvest, with accessory bands arising from the STT being particularly common. These bands may insert into a variety of fascial structures, including the gastrocnemius fascia, tibial periosteum, and, less frequently, the sartorial or soleus fascia [5,8,9].

In a cadaveric study of 102 knees from a caucasian population, Olewnik et al. reported that 47.1% of specimens demonstrated at least one accessory band arising from the STT [9]. A similar study in an Asian population by Tanpowpong et al. examined 80 knees and identified even greater anatomical variability, with 94% of specimens exhibiting at least one accessory band arising from the STT and 80% demonstrating at least one arising from the GT [5].

In contrast, accessory bands arising from the sartorius are rarely reported in the literature and, when present, are typically accompanied by accessory bands from the STT, the GT, or both [9]. The present findings therefore raise the possibility of a rare sartorial accessory band occurring in the absence of additional STT or GT bands, which does not fit into a current classification system.

Although the additional structure observed in this case appeared to course anteriorly, a limitation of this case is that definitive proximal tracing to a muscle belly was not performed, and the precise origin of the structure cannot be confirmed based on intraoperative findings alone. Alternatively, the structure may represent an accessory band arising from the semitendinosus tendon (STT) and inserting into deeper layers of the sartorial fascia, a configuration that has been described in previous anatomical studies [4,5]. Furthermore, postoperative imaging was not performed to further characterise the structure’s course. As a single intraoperative observation, this case alone cannot be used to define a new anatomical classification.

However, a sartorial origin remains a possibility, as the accessory tendon showed no connection with the STT or the GT. During harvesting of the STT and GT, no continuity with either tendon was observed, and the accessory tendon remained intact after both tendons were harvested. Furthermore, upon minimal tracing, it was found to course toward the anterior compartment of the thigh.

## Conclusions

This case highlights an unusual intraoperative finding at the pes anserinus of an additional tendon-like structure of uncertain origin encountered following confirmed harvest of the gracilis and semitendinosus tendons, though the available intraoperative findings do not allow its anatomical origin or classification to be established definitively.

Regardless of its origin, if an unidentified structure is encountered during hamstring tendon harvest, surgeons should proceed with caution and adopt a methodical approach to accurately identify all relevant anatomical structures before dividing any unrecognized structure, ensuring that the semitendinosus and gracilis tendons are harvested appropriately while preserving any accessory bands. Failure to do so risks incomplete graft harvest or inadvertent injury to the primary tendons. Postoperative MRI could be considered in future cases to allow non-invasive proximal tracing and better characterise such structures. Future studies incorporating detailed intraoperative documentation, cadaveric dissection and postoperative imaging may help establish the origin, prevalence and classification of similar tendon-like structures.

## References

[1] Curtis BR, Huang BK, Pathria MN, Resnick DL, Smitaman E (2019). Pes anserinus: anatomy and pathology of native and harvested tendons. AJR Am J Roentgenol.

[2] Paschos NK, Howell SM (2016). Anterior cruciate ligament reconstruction: principles of treatment. EFORT Open Rev.

[3] Candal-Couto JJ, Deehan DJ (2003). The accessory bands of gracilis and semitendinosus: an anatomical study. Knee.

[4] Yasin MN, Charalambous CP, Mills SP, Phaltankar PM (2010). Accessory bands of the hamstring tendons: a clinical anatomical study. Clin Anat.

[5] Tanpowpong T, Saengkhiew C, Huanmanop T, Itthipanichpong T (2019). Anatomical variations of accessory bands in semitendinosus and gracilis tendons among the Asian population. Asian J Sports Med.

[6] Charalambous CP, Kwaees TA (2012). Anatomical considerations in hamstring tendon harvesting for anterior cruciate ligament reconstruction. Muscles Ligaments Tendons J.

[7] Lobos Centeno EA, Martinel V, Cassinelli JE, Colombet P, Barth J (2025). Optimizing hamstring tendon graft harvesting technique for anterior cruciate and anterolateral ligament reconstruction. Arthrosc Tech.

[8] Olewnik Ł, Podgórski M, Polguj M (2020). An unusual insertion of an accessory band of the semitendinosus tendon: case report and review of the literature. Folia Morphol (Warsz.

[9] Olewnik Ł, Gonera B, Podgórski M, Polguj M, Jezierski H, Topol M (2019). A proposal for a new classification of pes anserinus morphology. Knee Surg Sports Traumatol Arthrosc.

